# Risk Factors and Spatial-Temporal Analysis of Porcine Reproductive and Respiratory Syndrome Seroprevalence in China Before and After African Swine Fever Outbreak

**DOI:** 10.3389/fvets.2022.929596

**Published:** 2022-08-02

**Authors:** Pengfei Zhao, Chaofei Wang, Wenjian Cao, Rui Fang, Junlong Zhao

**Affiliations:** State Key Laboratory of Agricultural Microbiology, College of Veterinary Medicine, Huazhong Agricultural University, Wuhan, China

**Keywords:** risk factors analysis, spatial-temporal analysis, seroprevalence, PRRSV, ASF outbreak

## Abstract

Porcine reproductive and respiratory syndrome (PRRS) is an infectious viral disease that causes great harm to the pig industry. PRRS virus (PRRSV), the causative agent of PRRS, is characterized by severe reproductive failure and respiratory confusion. This study performed a cross-sectional investigation of PRRSV seroprevalence and collected 14,134 serum samples in pig farms without PRRSV vaccination from 12 provinces and two cities in China from 2017 to 2021 to detect PRRSV antibodies by enzyme-linked immunosorbent assay (ELISA). The apparent and true PRRSV antibody prevalence was estimated and compared based on the Clopper-Pearson method and Pearson chi-square test, respectively. Risk factors associated with the PRRSV serological status of pig farms were analyzed through univariate and multivariable logistic regression analysis. An automatic autoregressive integrated moving average (ARIMA) model procedure was used for time-series analysis for PRRSV seroprevalence. Spatial clusters of high PRRSV seroprevalence were detected by SaTScan software. The total true PRRSV seroprevalence of the animal level was 62.56% (95% confidence interval [CI]: 61.74–63.37%). Additionally, 286 out of 316 pig farms were positive for PRRSV antibodies at the herd level. Pig farms without pseudorabies virus (PRV) infection were 5.413 (95% CI: 1.977–17.435) times more likely to be PRRSV antibody positive than those with PRV. Identically, the possibility of pig farms being PRRSV antibody positive before an African swine fever (ASF) outbreak was 3.104 (95% CI: 1.122–10.326) times more than after ASF. The odd ratio values of medium and large pig farms with PRRSV infection are 3.076 (95% CI: 1.005–9.498) and 6.098 (95% CI: 1.814–21.290). A fluctuant decline pattern for PRRSV prevalence was observed in the temporal analysis. Three significant clusters of high PRRSV seroprevalence were first detected in China, covering a time frame from January 2018 to September 2018, which reveals high PRRSV prevalence before the outbreak of ASF. These findings show the epidemic situation and spatial-temporal distribution of PRRSV infection in China in recent years and could help develop reasonable measures to prevent PRRSV infection.

## Introduction

Porcine reproductive and respiratory syndrome (PRRS) is an infectious viral disease that is caused by PRRSV and characterized by reproductive failure in sows and severe respiratory confusion and mortality in young pigs. It is responsible for substantial economic losses in the global swine industry (1–3). PRRSV is a single-stranded, positive-sense RNA virus from *Arteriviridae*, genus *Arterivirus*, that contains ≈15 kb nucleotides and encodes >10 open reading frames (ORFs) (4, 5). PRRSV had two main genotypes, namely European type (type 1) and North American type (type 2), of which North American type is the major epidemic genotype in China (6–9).

The first PRRSV strain of China was isolated and identified from aborted fetuses in 1996 that belonged to the North American type (8, 10). A highly pathogenic PRRSV (HP-PRRSV) strain that was featured with the discontinuous deletion of 30 amino acids in the Nsp2 gene and 20% mortality in pigs had occurred and resulted in the deaths of one million pigs in China in 2006 (11, 12). In recent years, new PRRSV field isolates with low mortality show high similarity in gene sequences with NADC30 strains isolated in America in 2008 and have been successively reported and defined as NADC30-like strains in China since 2014 (13, 14). The HP-PRRSV and NADC30-like strains are the primary epidemic strains that circulate in pig farms in China (9). Three hundred and sixty-five PRRSV strains were isolated by Jiang et al. (15) from 1996 to 2017 in China and used to analyze evolution and genome, which demonstrated that the HP-PRRSV, NADC30-like, and intermediated PRRSV were the major epidemic strains.

Infection and transmission of PRRSV from infected pigs to susceptible pigs can horizontally or vertically emerge through direct or indirect contact within the herd (6). Pitkin et al. even reported that PRRSV could be disseminated by aerosol with a distance of ≈120 m (16). The high variability in the PRRSV genome and diversity in PRRSV transmission modes causes tremendous challenges in controlling PRRSV infection in fields. Therefore, knowing the risk factors and spatial-temporal distribution of PRRSV infection is necessary to contain and eradicate PRRS illness.

Risks of PRRSV infection in pig herds increased with increased pig farm size, absence of purchased gilts quarantine, and semen purchased outside for artificial insemination (17). Fablet et al. (18) found that setting the temperature low in fattening rooms and with pigs, which coinfect with *Mycoplasma hyopneumoniae* and H1N2 swine influenza viruses, could raise herds' PRRSV seropositive possibility. Spatial analysis of PPRSV type 1 and 2 seroprevalences executed in Denmark statistically significantly detected clusters from 2007 to 2010 with higher PRRSV type 1 seroprevalence (19). However, related research on risk factors and spatial-temporal distribution of PRRSV infection are highly inadequate in China. Meanwhile, the ASF outbreak across China has caused a massive loss of pig populations, threatened the stability of the meat supply chain, and changed the feeding and management mode of pig farms, which is important for China's pig industry (20, 21). Therefore, knowing about the epidemic situation of PRRSV infection and providing reference information for policymakers related to future PRRSV control in China is urgently demanded after the ASF outbreak in 2018 (22).

## Materials and Methods

### Sampling Region and Population

This study included 14,134 serum samples from 316 farms without PRRSV vaccination located in 12 provinces and two cities in China from 2017 to 2021. Collated serum samples were tested for PRRSV antibodies using an enzyme-linked immunosorbent assay (ELISA). Then, attained data were used to seek risk factors associated with the PRRSV serological status of pig farms and investigate spatial-temporal PRRSV seroprevalence clusters in China before and after the ASF outbreak. The sampling regions successively covered all seven geographical areas of China, namely central (Henan, Hubei, and Hunan provinces), eastern (Shandong, Jiangsu, Anhui, Jiangxi, Fujian provinces, and Shanghai city), northeast China (Liaoning province), south (Guangdong province), southwest (Sichuan province), northwest (Shaanxi province), and northern (Tianjin city) districts. These areas are ≈2.3 million square kilometers located in longitudes of 97°20′ E to 126°00′ E and latitudes of 18°10′ N to 43°30′ N with high pig feeding density. There are various monsoon climates with 3–28°C of annual average temperature and multiple geographic patterns, including plateaus, mountains, plains, hills, and basins. Pigs were divided into six categories according to different age and usage as follows: piglets (from birth to 21 days), weaned piglets (age of 22–70 days), growing-finishing pigs (above 70 days), replacement gilts, multiparous sows (at least one party), and boars (23). All the selected pig farms were without PRRSV vaccine immunization. Moreover, location coordinates of pig farms were acquired from Baidu Map (https://map.baidu.com/).

### Sampling Design

Biosecurity measures were enhanced in pig farms after the ASF outbreak, thereby increasing the difficulty of collecting serum. Meanwhile, an accurate estimate for the number of pigs in the study region was inadequate. Therefore, a convenience sampling plan was conducted in this investigation. Detailed variable information on animal and farm levels was obtained by face-to-face interviews with pig farm owners. The documented variable information primarily included sampling time, sampling position, number of samples, season, farm size, pig farm topography, the background of pigs, ASF outbreak, and pseudorabies virus (PRV) purification in pig farms. The ASF outbreak, PRV purification, and pig farms topography are binary variables. Farm size is defined as small (≤100 sows), medium (100–500 sows), and large (≥500 sows) according to the number of sows in herds.

### Sampling Method

Randomly selected pigs had blood extracted from the precaval vein using sterile vacutainer tubes without decoagulant. The gathered whole blood was sent to a third-party laboratory, “Wuhan Keweichuang Biotechnology Co, Ltd,” in a cold chain and centrifuged to obtain serum under 3,000 rpm for 5 min. The acquired serum was stored at −20°C until use.

### Detection of Serum for PRRSV Antibody by ELISA

PRRSV antibodies were detected by the PRRS X3 Ab Test kit with a sensitivity of 98.8% and specificity of 99.9% (IDEXX, USA) according to instruction (24). The operating procedures were described as follows. First, the serum was diluted in a ratio of 1:40 by sample diluent. Then 100 μl of diluted serum was added to the coated plates and incubated for 30 min at 18–26°C. Secondly, the plates' solution was discarded, and the plates were washed three times using the wash solution. Subsequently, 100 μl of the conjugate was added to plates to incubate for 30 min at 18–26°C again. The above-mentioned wash process was repeated. Thirdly, 100 μl of the substrate was dispensed to test the well for 15 min of incubation in a dark place. Then 100 μl of stop solution was added to the wells. Finally, the absorbance of each well in plates was measured at 650 nm by Multiskan FC (Thermo scientific, USA). The S/P value of PRRSV antibodies was calculated according to the formula: (absorbance of sample-absorbance of negative control)/(absorbance of positive control-absorbance of negative control). S/P values ≥ 0.4 were considered PRRSV antibody positive. Otherwise, the serum was negative. A pig farm was deemed as PRRSV infection with at least a PRRSV antibody-positive sample.

### Data Analysis

Obtained data were entered and organized in Excel (Microsoft Excel 2007, USA). The apparent and true PRRSV prevalence of animal levels was estimated using the EpiR package (version 2.0.43) based on the Clopper-Pearson method (25). Simultaneously, the Pearson chi-square test was used to analyze the differences in PRRSV seroprevalence among provinces and pig categories (26).

The serological status of pig farms was registered as a dichotomous variable (positive or negative). The potential risk factors associated with the serological status of pig farms were explored among putative variables using univariate logistic regression analysis. Variables with a *p*-value of <0.1 in the univariate analysis were selected for multivariable logistic regression analysis (27). The variance inflation factor (VIF) was used to identify multicollinearity (28). Variables relevant to biological meaning are retained in the model in presence of multicollinearity between variables.

Samples were not collected because of coronavirus disease 2019, which caused missed PRRSV seroprevalence data in February and March 2020. Hence, the missed data were filled using the mice package (version 3.14.0) through multiple interpolation methods (29). Time-series of PRRSV seroprevalence were analyzed using an automatic (ARIMA) procedure. The parameters p (the number of autoregressive terms), d (the number of non-seasonal differences), and q (the number of moving average terms) were determined to forecast PRRSV seroprevalence with 95 and 80% CI in the 19 months following the study period (May 2021).

SaTScan software release 9.6 version was employed to analyze spatial and temporal clusters of high PRRSV seroprevalence (30). The numbers of PRRSV seropositive and seronegative samples from each pig farm were respectively treated as case and control groups. We used the month level for time aggregation to cover all PRRSV seropositive samples from December 2017 to May 2021. All data analyses were achieved utilizing R software (31). Maps were plotted using ArcGIS 10.7 (ESRI, USA).

## Result

### PRRSV Seroprevalence of Animal and Herd Levels

**Figure 1:**
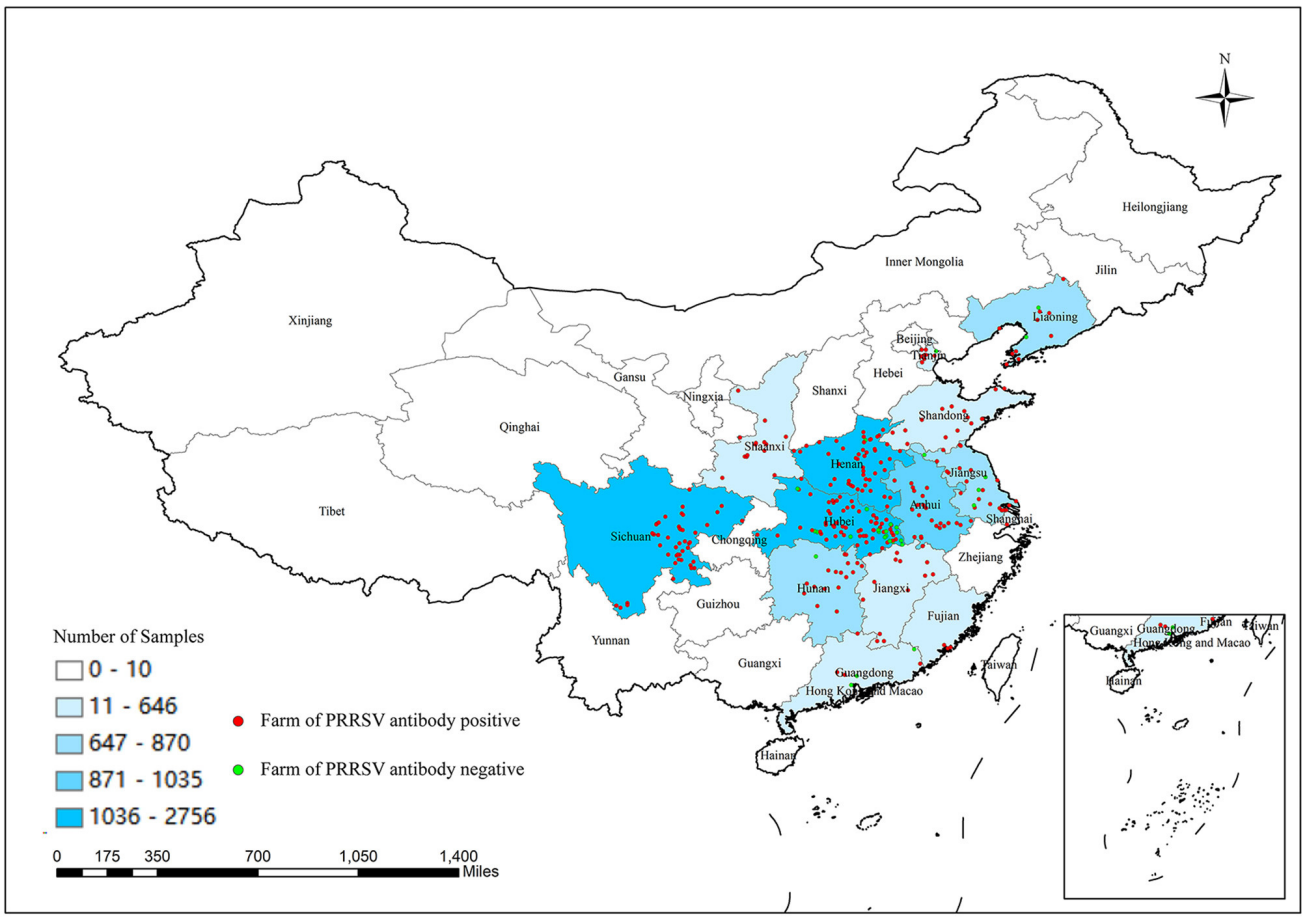
Number of samples collected from different provinces or cities in China and location of selected pig farms.

**Figure 2:**
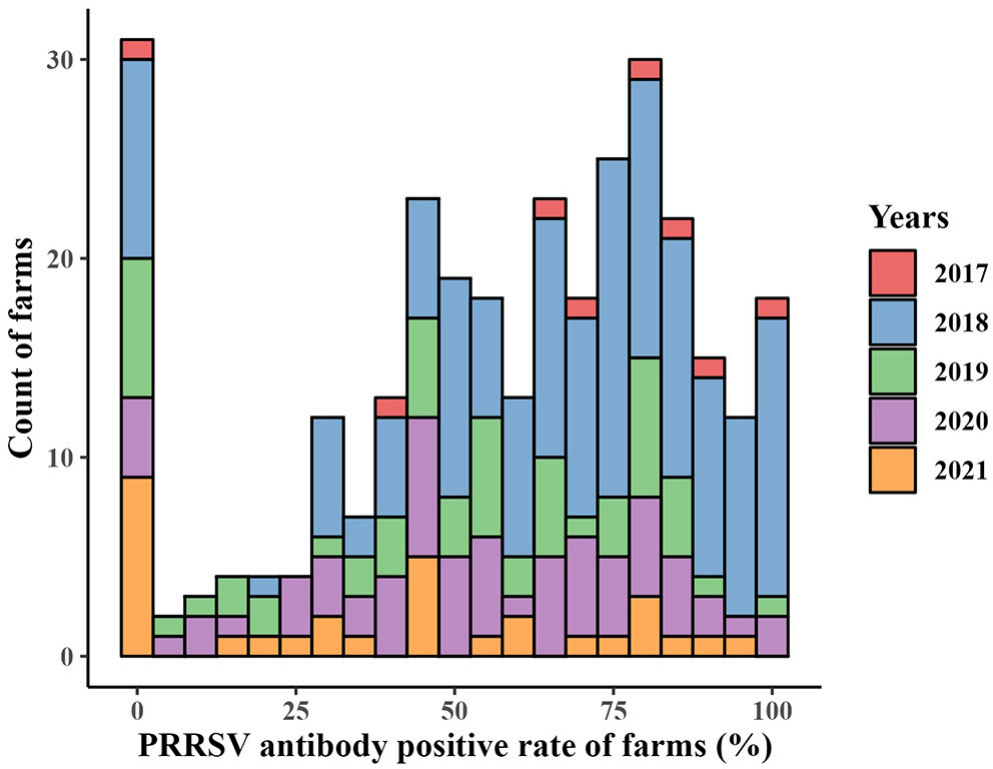
Histogram of PRRSV prevalence of pig farms from December 2017 to May 2021.

The numbers of collected samples in each province and locations of pig farms are shown and labeled in Figure 1. A total of 14,134 samples were obtained with an overall 62.56% (95% CI: 61.74–63.37%) true prevalence of PRRSV infection. PRRSV seroprevalences of Tianjin city and Liaoning province have the highest (>80%). Hubei and Shaanxi provinces have the lowest PRRSV prevalence in all sampling regions (<50%). The Pearson chi-square test results revealed statistically significant differences in PRRSV prevalence in various provinces and pig categories (Tables 1, 2). The true prevalence of replacement gilts is the highest (75.466% 95% CI: 73.000–77.821%). The lowest prevalence of PRRSV (43.304%, 95% CI: 41.400–45.228%) appears in weaned-piglets. We selected 316 pig farms to sample serum in this study, of which 286 are PRRSV antibody-positive and 30 pig farms PRRSV antibody-negative (data not shown). Figure 2 shows the histogram of PRRSV antibody positive rate of pig farms (mean: 58.12%; median: 64.69%; range: 0–100%).

**Table 1:** The apparent and true prevalence of PRRSV antibody with Pearson chi-square test in 12 provinces and two cities in China.

**Provinces**	**No. of positive samples**	**No. of total samples**	**Apparent prevalence with 95% CI (%)**	**True prevalence with 95% CI (%)**	**χ^2^ values**	***P* value**
Anhui	676	1,035	65.31 (62.33–68.22)	66.07 (63.05–69.01)	814.260	<2.2 × 10^−16^^***^
Fujian	135	215	62.79 (55.96–69.27)	63.52 (56.59–70.08)		
Guangdong	133	193	68.91 (61.87–75.36)	69.72 (62.58–76.26)		
Henan	1,911	2,611	73.19 (71.45–74.88)	74.05 (72.29–75.77)		
Hubei	1,249	2,756	45.32 (43.45–47.20)	45.81 (43.92–47.72)		
Hunan	450	870	51.72 (48.35–55.09)	52.30 (48.88–55.72)		
Jiangsu	486	795	61.13 (57.64–64.54)	61.84 (58.30–65.29)		
Jiangxi	327	550	59.45 (55.22–63.59)	60.14 (55.84–64.32)		
Liaoning	604	743	81.29 (78.30–84.03)	82.26 (79.23–85.04)		
Shandong	421	646	65.17 (61.36–68.85)	65.93 (62.06–69.65)		
Shanghai	92	165	55.76 (47.83–63.47)	56.39 (48.36–64.21)		
Shaanxi	235	549	42.81 (38.62–47.06)	43.27 (39.03–47.58)		
Sichuan	1,584	2,471	64.10 (62.18–66.00)	64.85 (62.89–66.77)		
Tianjin	438	535	81.87 (78.34–85.04)	82.85 (79.27–86.06)		
Total	8,741	14,134	61.84 (61.04–62.65)	62.56 (61.74–63.37)		

*CI, confidence interval*.

^*^p < 0.05;

^**^p < 0.01;

*** *p < 0.001*.

**Table 2:** The apparent and true prevalence of PRRSV antibody with Pearson chi-square test in different stages of pigs.

**Background**	**No. of positive samples**	**No. of total samples**	**Apparent prevalence with 95% CI (%)**	**True prevalence with 95% CI (%)**	**χ^2^ values**	***P* value**
Piglets (≤21 days)	666	1,401	47.537 (44.894–50.191)	48.062 (45.384–50.751)	769.570	<2.2 × 10^−16^^***^
Weaned piglets (22–70 days)	1,149	2,682	42.841 (40.958–44.740)	43.304 (41.400–45.228)		
Growing-finishing pigs (≥71 days)	1,366	1,953	69.944 (67.856–71.972)	70.764 (68.648–72.818)		
Replacement gilts	989	1,326	74.585 (72.151–76.910)	75.466 (73.000–77.821)		
Multiparous sows (≥1 parity)	3,360	4,838	69.450 (68.130–70.746)	70.264 (68.927–71.577)		
Boar	1,211	1,934	62.616(60.416–64.778)	63.340(61.111–65.530)		
Total	8,741	14,134	61.84 (61.04–62.65)	62.56 (61.74–63.37)		

*CI, confidence interval*.

^*^p < 0.05;

^**^p < 0.01;

*** *p < 0.001*.

### Risk Factors Analysis Related to PRRSV Serological Status of Pig Farms

Season and topography variables are not significantly associated with the PRRSV serological status of pig farms (*p*-value > 0.1) in the univariate logistic analysis presented in Table 3, which are removed from the subsequent multivariable logistic analysis. The identified risk factors associated with the PRRSV serological status of pig farms are shown in Table 4 through multivariable logistic analysis. Medium and large pig farms are 3.076 (95% CI: 1.005–9.498) and 6.098 (95% CI: 1.814–21.290) times more likely to be PRRSV antibody-positive compared with small pig farms, respectively. Similarly, the possibility of pig farms being PRRSV antibody-positive before the ASF outbreak is 3.104 (95% CI: 1.122–10.326) times that after the ASF outbreak. The odds ratio (OR) value of pig farms with PRV purification is 5.414 (95% CI: 1.977–17.435), implying that PRV purification may decrease PRRSV infection pressure.

**Table 3:** Univariate logistic analysis of risk factors associated with PRRSV serological status of pig farm levels.

**Variables**	**Category**	**OR with 95% CI**	***P* value**
Season	Autumn	1 (Reference)	
	Spring	0.704 (0.436–3.024)	0.767
	Summer	1.439 (0.508–5.315)	0.449
	Winter	0.793 (0.575–5.242)	0.349
Size	Small (<100 sows)	1 (Reference)	
	Medium (100–500 sows)	3.655 (1.378–9.429)	7.576 × 10^−3^^**^
	Large (>500 sows)	5.805 (2.037–16.911)	9.630 × 10^−4***^
Geographic location of pig farm	Eastern China	1 (Reference)	
	Central China	0.290 (0.0668–0.880)	0.051
	North China	0.455 (0.0520–9.683)	0.512
	Northeast of China	0.205 (0.0297–1.708)	0.105
	Northwest of China	1.429 (0.0366–3.145)	0.996
	South China	0.756 (0.0101–0.496)	0.006**
	Southwest of China	1.429 (0.0716–1.741)	0.991
Pig farm topography	Hill or mountain	1 (Reference)	
	Plain	2.118 (0.890–5.869)	0.113
After the ASF outbreaks	Yes	1 (Reference)	
	No	2.772 (1.113–8.407)	0.044*
PRV purification	No	1 (Reference)	
	Yes	3.475 (1.515–8.993)	0.005**

*OR, odds ratio*.

^*^p < 0.05;

^**^p < 0.01;

*** *p < 0.001*.

**Table 4:** Multivariable logistic analysis of risk factors associated with PRRSV serological status of pig farm levels.

**Variables**	**Category**	**OR with 95% CI**	***P* value**
Size	Small (<100 sows)	1 (Reference)	
	Medium (100–500 sows)	3.076 (1.005–9.498)	0.048^*^
	Large (>500 sows)	6.098 (1.814–21.290)	0.004^**^
Geographic location	Eastern China	1 (Reference)	
	Central China	0.496 (0.107–1.699)	0.306
	North China	0.215 (0.021–4.924)	0.226
	Northeast of China	0.137 (0.016–1.351)	0.068
	Northwest of China	3.219 (0.061–4.526)	0.995
	South China	0.113 (0.013–0.956)	0.042^*^
	Southwest of China	2.416 (0.019–8.758)	0.991
After the ASF outbreaks	Yes	1 (Reference)	
	No	3.104 (1.122–10.326)	0.042^*^
PRV purification	No	1 (Reference)	
	Yes	5.413 (1.977–17.435)	0.002^**^

*OR, odds ratio*.

* *p <0.05*;

** *p <0.01*;

^***^p < 0.001.

### Temporal Analysis of PRRSV Seroprevalence

**Figure 3:**
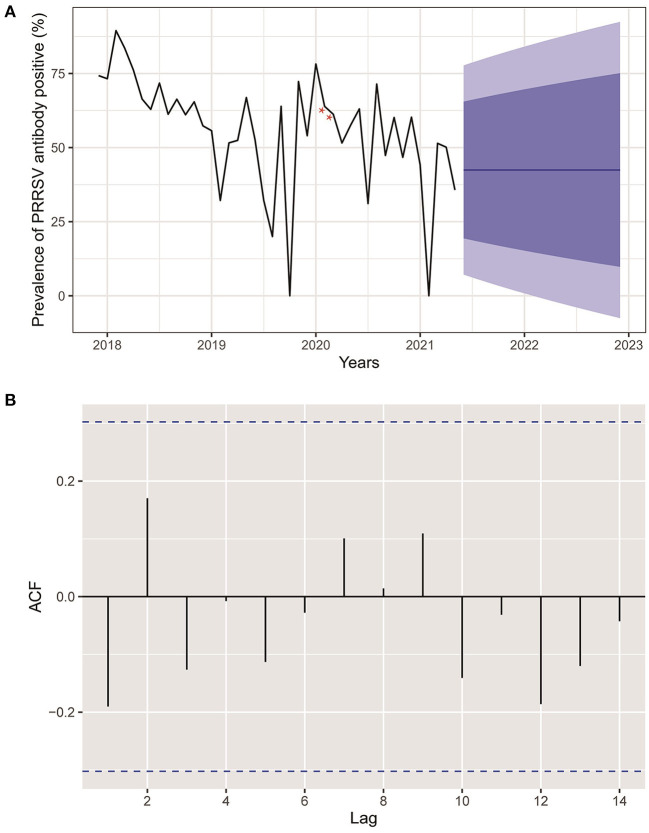
**(A)** Actual and forecasted PRRSV prevalence. Black line was the actual PRRSV prevalence from December 2017 to May 2021. The blue line represented the forecasted PRRSV prevalence in the next 19 months. The dark and light blue areas were 80 and 95% CI of forecasted PRRSV prevalence, respectively. Red stars indicated interpolated values for PRRSV prevalence of February and March 2020 deficiency because of COVID-2019. **(B)** Autocorrelation plots of PRRSV prevalence.

Figure 3A reveals an epidemic curve of PRRSV seroprevalence by month. The highest PRRSV prevalence appeared in February 2018 and showed a comprehensive fluctuant decline trend since November 2018. Meanwhile, an ARIMA model has been established and used to forecast PRRSV prevalence with predictive limits of 95 and 80% CI in the 19 months following the current research date (May 2021). No change pattern of autocorrelation residuals was observed, which indicated the applicability of the established model and forecasts of PRRSV prevalence in this study (Figure 3B).

### Spatial Analysis of PRRSV Seroprevalence

**Figure 4:**
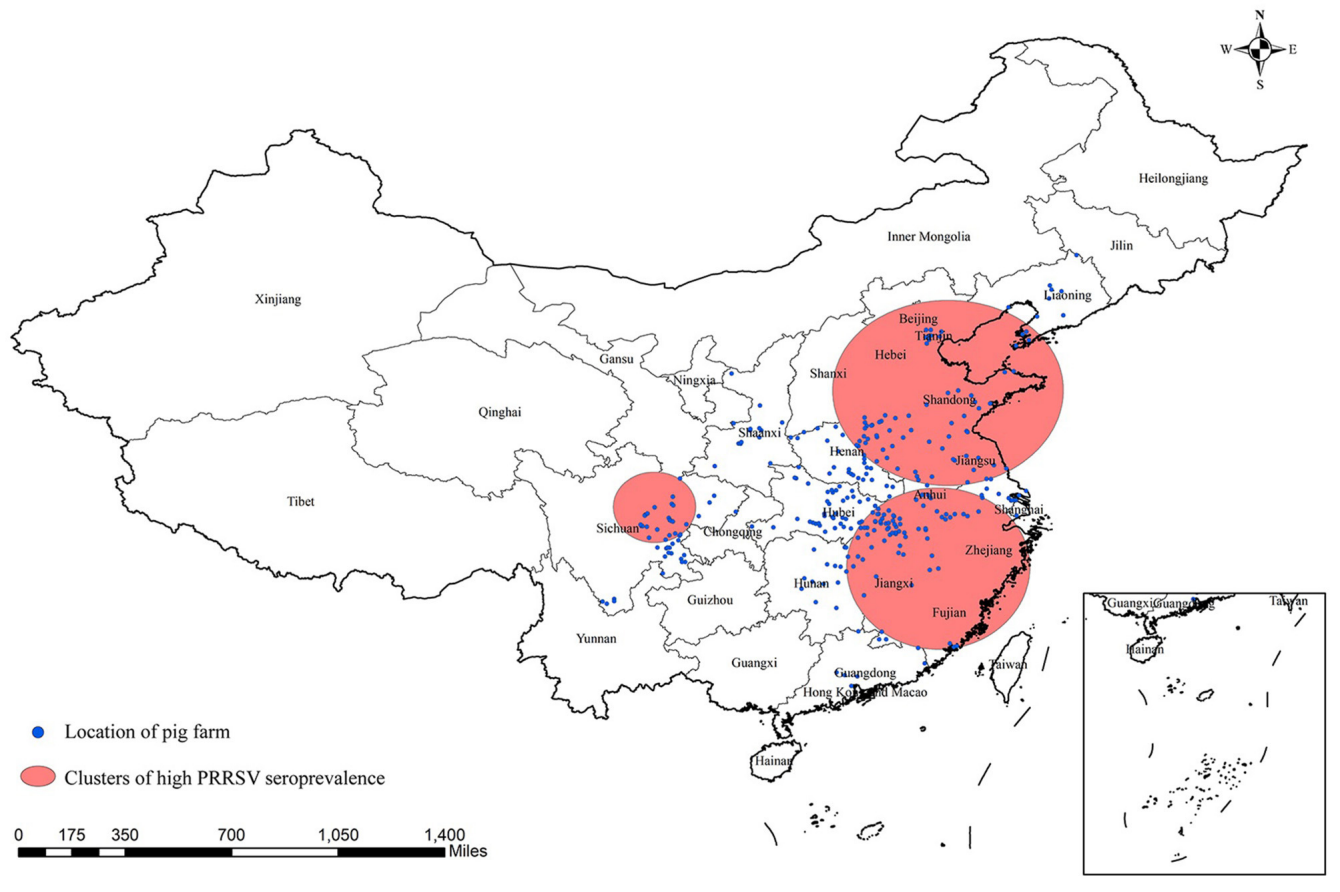
Significant spatial clusters (*p*-value < 0.05) of high PRRSV seroprevalence in China from December 2017 to May 2021 with a maximum window size of 25% of the population at risk.

Three significant clusters of high PRRSV seroprevalence are detected in China through spatial-temporal analysis from December 2017 to May 2021 (Figure 4, Table 5). The time frame of three clusters of high PRRSV seroprevalence all occurred between January and September 2018, before the ASF outbreak. The coordinate of the largest cluster is 36.657313 N, 118.008476 E, with a radius of 489.26 km. Its time frame and relative risk are from January 1, 2018, to September 30, 2018, and 1.37, respectively. The second cluster locates at 28.306173 N, 117.546305 E, with a radius of 426.62 km. This cluster's time frame and relative risk are from March 1, 2018, to March 31, 2018, and 1.58, respectively. The coordinate of the smallest cluster is 31.220567 N, 104.046397 E, with a radius of 186.47 km. The time frame and relative risk are January 1, 2018, to May 31, 2018, and 1.54, respectively.

**Table 5:** Spatial-temporal analysis of PRRSV seroprevalence in China from 2017 to 2021.

**Cluster**	**Coordinates**	**Radius (km)**	**Time frame**	**Relative risk**	***P* value**
1	36.657313 N,118.008476 E	489.26	2018/1/1–2018/9/30	1.37	<10^−16^^***^
2	28.306173 N,117.546305 E	426.62	2018/3/1–2018/3/31	1.58	<10^−16^^***^
3	31.220567 N, 104.046397 E	186.47	2018/1/1–2018/5/31	1.54	<10^−16^^***^

^*^p < 0.05;

^**^p < 0.01;

*** *p < 0.001*.

## Discussion

PRRSV is the causative agent of PRRS and is an influential pig disease that causes great harm to the pig industry. Meanwhile, China is the largest pork producer and consumer worldwide (32), thus, investigating the epidemic status of PRRSV infection in China is necessary. However, most studies about PRRSV epidemiology focused more on molecular genetic evolutionary analysis in China, not on serological prevalence (15, 33–35). Consequently, we performed a widespread cross-sectional study to collect serum samples in pig farms without PRRSV vaccination from 12 provinces and two cities in China from 2017 to 2021. Implementing a complete sampling plan, including most pig farms in China, is impracticable because of the inability to know accurate numbers of pigs fed in China and the expense limit. Therefore, convenience sampling was adopted in our study.

A total of 14,134 serum samples were collected from 316 pig farms to detect antibodies by ELISA as PRRSV infection diagnosis. The PRRSV seroprevalence of animal and herd levels are, respectively, 61.84 and 90.51% (286 pig farms of PRRSV seropositive), implying a high PRRSV infection in China, which follows a previous report performed by Guo et al. (9), wherein >80% of pig farms were PRRSV seropositive. The apparent and true prevalence of PRRSV infection in different provinces and pig categories show subtle differences, which indicates our results can reflect real PRRSV infection situations. Meanwhile, a significant difference was found in PRRSV seroprevalence for diverse provinces or cities by the Pearson chi-square test, which imply a probable existence of different spatial risks of PRRSV infection. The top three highest PRRSV seroprevalences are Tianjin, Liaoning, and Henan, all located to the north of China. The reason for this might be the low temperature that contributes to PRRSV survival and increases infection risk (36). The PRRSV prevalence in growing-finishing pigs, replacement gilts, boars, and multiparous sows are all >60%, which signifies that they could be a major source of PRRSV infection in herds. The replacement gilts had the highest PRRSV prevalence of 75.466% and are a dangerous signal for comprehensive control of PRRSV in herds because gradually replacing multiparous sows with negative gilts has been an important measure for PRRSV eradication (37). Previous research has also reported that quarantining new incoming sows can reduce the risks of PRRSV infection (38). Maternal antibodies of PRRSV obtained from sows can maintain for ≈2–4 weeks in piglets and start to decline at 4–10 weeks of age (39). Hence, the PRRSV prevalence of piglets and weaned-piglets might be overestimated because of the existence of maternal antibodies. Meanwhile, we can infer that the PRRSV positive antibodies of growing-finishing are induced by field PRRSV infection because the maternal antibodies have disappeared at this stage.

The possibility of pig farms being PRRSV seropositive increased with the size of pig farms, as already described by Firkins et al. (17), who revealed an association between larger herd size with increased risks of PRRSV infection. The farmers of pig farms without PRRSV vaccination often prefer to control disease through enhanced biosecurity measures whether on large or small farms, which implies that biosecurity levels might be similar for pig farms of different sizes (private communication). However, the high probability of PRRSV seropositivity in large pig farms might be due to the following reasons. More frequent contact between pigs in large pig farms might enhance the chances of virus spread. Furthermore, pigs on small pig farms have a simpler herd structure and can receive more care from breeders. A pig farm with PRV infection was 3.104 times more likely to be PRRSV-positive than one without PRV infection, which implies that PRV infection in pig herds was associated with a PRRSV seropositive status, playing a similar role in PRRSV infection as *Mycoplasma hyopneumoniae* and H1N2 swine influenza A virus found by Fablet et al. (18). The underlying mechanisms and causal relationship of these viral interactions remained murky, hinting that control of PRRSV infection needed to simultaneously adopt collaborative measures for multiple viruses in herds. The OR value of pig farms being PRRSV seropositive before the ASF outbreak was remarkably higher than that after the ASF outbreak, which was potentially caused by implementation of strengthened biosafety measures in pig farms after the ASF outbreak (40). Only the OR value of pig farms located in south China was statistically significant compared to the reference pig farms located in eastern China in the geographic location variable. However, considering the large *p*-value (0.042) and possible sampling error, the geographic location variable might not be the risk factor related to the PRRSV serological status of farms.

Little information is available about the spatial-temporal distribution of PRRSV infection in China due to the weak development of veterinary epidemiology, not to mention the comparation of the PRRSV epidemic before and after the ASF outbreak. Additionally, spatial-temporal analysis of PRRSV infection can contribute to detecting clusters of high PRRSV prevalence and exploring variation trends of PRRSV infection helping policymakers to design more precise and cost-effective intervention policies related to future PRRSV control in China. This study was the first to perform a spatial-temporal analysis of PRRSV seroprevalence in China after the ASF outbreak. The prevalence of PRRSV began a gradual decline in November 2018, when ASF entered China. The results of temporal analysis of PRRSV prevalence in our study exhibited a fluctuant decline pattern without obvious seasonal or periodic trends, which might be caused by the disadvantage of convenience sampling method with imperfect sample representativeness, although the incidence of PRRSV infection in autumn and winter is usually higher than that in spring and summer according to expert opinions (private communication) (41). Additionally, the PRRSV prevalence by month in Figure 3 showed a general decline after the ASF outbreak similar to the risk factor analysis described above. The forecasted values of PRRSV prevalence in the next 19 months (until December 2022) remained unchanged, which indicated that the PRRSV prevalence would tend toward a stable epidemic in China in the future. Three significant-high PRRSV seroprevalence clusters were first detected in China: two large clusters located in eastern China and a small cluster located in southwest China close to Sichuan province. Interestingly, the time frames of the three clusters of high PRRSV seroprevalence were all between January 2018 and September 2018 before the massive ASF outbreak in China (42), further demonstrating reduced PRRSV infection after the ASF outbreak. The detailed spatial epidemiology of PRRSV infection in China remained unknown, which demands another example to study, although we detected three clusters of high PRRSV prevalence in this study. We all demonstrate that PRRSV seroprevalence after the ASF outbreak displays an apparent decrease compared to that before the ASF outbreak through risk factors and spatial-temporal analysis.

We collected 14,134 samples from 316 pig farms without PRRSV vaccination located in 12 provinces and two cities in China from 2017 to 2021 to detect PRRSV antibodies using the ELISA method. The total true prevalence rate of PRRSV was 62.56% (95% CI: 61.74–63.37%) for pig level, and 286 pig farms (90.51%) were PRRSV antibody-positive, showing a widespread PRRSV epidemic in China. Additionally, we found that farm size, the ASF outbreak, and PRV purification variables were risk factors associated with the PRRSV serological status of pig herd using the multivariable logistic analysis. Temporal analysis for PRRSV seroprevalence showed a fluctuant declining trend. Three significant clusters of high PRRSV seroprevalence that occurred before the ASF outbreak were first detected in China through spatial-temporal analysis. The research findings obtained in our study fill in the knowledge gap of the epidemic situation, risk factors, and spatial-temporal distribution of PRRSV infection in China in recent years and could help form policies for PRRSV prevention in the future.

## Data Availability Statement

The raw data supporting the conclusions of this article will be made available by the authors, without undue reservation.

## Ethics Statement

The animal study was reviewed and approved by the Ethical Committee of Huazhong Agricultural University Huazhong Agricultural University. Written informed consent was obtained from the owners for the participation of their animals in this study.

## Author Contributions

Conceptualization, experimental design, and project guide: JZ. Investigation, sampling, data analysis, and writing draft: PZ. Investigation and sampling: CW and WC. Supervision: RF. All authors have read and consented to publish the manuscript.

## Funding

This work was supported by the Fundamental Research Funds for the Central Universities in China (Project number: 2662020DKPY016).

## Conflict of Interest

The authors declare that the research was conducted in the absence of any commercial or financial relationships that could be construed as a potential conflict of interest.

## Publisher's Note

All claims expressed in this article are solely those of the authors and do not necessarily represent those of their affiliated organizations, or those of the publisher, the editors and the reviewers. Any product that may be evaluated in this article, or claim that may be made by its manufacturer, is not guaranteed or endorsed by the publisher.
